# Differentiation of Mesenchymal Stem Cells to Neuroglia: in the Context of Cell Signalling

**DOI:** 10.1007/s12015-019-09917-z

**Published:** 2019-09-12

**Authors:** Sajan George, Michael R. Hamblin, Heidi Abrahamse

**Affiliations:** 1 Laser Research Centre, University of Johannesburg, P.O. Box 17011, Doornfontein, 2028 South Africa; 2 Wellman Centre for Photomedicine, Massachusetts General Hospital, Boston, MA 02114 USA; 3 Department of Dermatology, Harvard Medical School, Boston, MA 02115 USA

**Keywords:** Differentiation, Glia, Neurons, Neurospheres, Organoids, Signalling, Stem cells

## Abstract

The promise of engineering specific cell types from stem cells and rebuilding damaged or diseased tissues has fascinated stem cell researchers and clinicians over last few decades. Mesenchymal Stem Cells (MSCs) have the potential to differentiate into non-mesodermal cells, particularly neural-lineage, consisting of neurons and glia. These multipotent adult stem cells can be used for implementing clinical trials in neural repair. Ongoing research identifies several molecular mechanisms involved in the speciation of neuroglia, which are tightly regulated and interconnected by various components of cell signalling machinery. Growing MSCs with multiple inducers in culture media will initiate changes on intricately interlinked cell signalling pathways and processes. Net result of these signal flow on cellular architecture is also dependent on the type of ligands and stem cells investigated in vitro. However, our understanding about this dynamic signalling machinery is limited and confounding, especially with spheroid structures, neurospheres and organoids. Therefore, the results for differentiating neurons and glia in vitro have been inconclusive, so far. Added to this complication, we have no convincing evidence about the electrical conductivity and functionality status generated in differentiating neurons and glia. This review has taken a step forward to tailor the information on differentiating neuroglia with the common methodologies, in practice.

## Introduction

Stem cells are bestowed with characteristics of perpetual growth, multiplication and the potential to differentiate into various cell types, tissues and even bodily organs. During developmental stages, Neural Stem Cells (NSCs) residing in the subventricular zone of the forebrain generate new neurons. Likewise, NSCs in the subgranular zone located in the hippocampus generate a small proportion of astroglia (1). Since, NSCs have only a limited ability for the regeneration of neuroglia in adult mammalian brain, stem cells will be a convenient option for replacement therapies during diseases or disorders. Adult stem cells from mesoderm have the potential to differentiate into neuronal and glial cells upon treatment with various inducers of growth. These Mesenchymal Stem Cells (MSCs) are capable of tissue repair by differentiating into adipocytes, chondrocytes and osteoblasts. They are also widely explored for their differentiation potential into endodermal lineages viz. cardiomyocytes, hepatocytes as well as ectodermal lineage, especially neuronal-lineage cells (2).

Differentiation of MSCs to functional neurons and glia seems to be a convenient option for the replacement therapies of neurodegenerative diseases and disorders (3, 4). Based on the mechanisms suggested by basic experiments and clinical trials, these cells hold promise for treating neurological maladies viz. Alzheimer’s disease, amyotrophic lateral sclerosis, cerebral palsy and Parkinson’s disease (5, 6). These diseases of the Central Nervous System (CNS) are an outcome of neuronal malfunctions arising due to the alterations in cellular signalling or metabolic events leading to cognitive dysfunction and paralysis. MSCs exert autocrine or paracrine effects for the replacement of genes or proteins in functionally impaired neuroglia, although, their ability to cross the blood-brain barrier is still under debate (7, 8). The most common types of neurological trauma are spinal cord injury and stroke due to accidents and cerebral hemorrhages, respectively. Although, there is little evidence that infused MSCs are differentiating to functional neurons, these multipotent cells enhance angiogenesis and migration of host neurogenic cells at the site of injury. Further, they secrete soluble paracrine factors to reduce the permeability of endothelium for innate as well as adaptive immune system (9, 10).

MSCs are abundant in adipose tissues, amniotic fluid, bone marrow and dental pulp, although, found scanty in endometrium, muscle, periosteum, placenta, synovial fluid and Wharton’s jelly (11). Although, harvest of MSCs from adipose tissue is less invasive than its extraction from bone marrow, the latter is widely used for cell therapies. Thus, various protocols have been developed using chemical or biological inducers using adipose- or bone marrow-derived MSCs for neuronal differentiation in vitro (12). Ikegame et al. (2011) showed that the neuronal differentiation potential of Adipose Stem Cells (ASCs) is higher than the MSCs from bone marrow, with a better outcome in an animal stroke model (13). Another study identified that following differentiation, MSCs from Wharton’s jelly and bone marrow had similar levels of expression of dopaminergic markers and neurotransmitter release (14). Besides, MSCs isolated from spleen and thymus show same capacity for differentiation to peripheral glia as those from bone marrow in a co-culture system (15).

MSCs hold the potential for differentiation into neuronal and glial cells, although, investigations are still in progress for identifying a suitable protocol for implementation in the clinical settings. In fact, the differentiation potential of MSCs is stimulated by cerebrospinal fluid or a conditioned media from glia indicating that the vital elements required for the differentiation of neuroglia may be lacking in a synthetic media (16, 17). Thus, for achieving a successful differentiation of MSCs to neuroglia, one must adjust the cell culture to conditions in situ*.* MSCs are extensively been experimented using a wide-range of growth inducers for neuronal differentiation. Often, the morphological and functional properties of differentiating MSCs are linked to changes due to the absorption and secretion of media components. Maturation of these progenitor cells to functional neuroglia may require tweaking of signalling processes by various inducers of differentiation for simulating in vivo conditions. Below is a summary of differentiating MSCs to neurons as well as glia in the context and complicity of various small molecules and signalling pathways.

## Cell Signalling

### Differentiation of Neurons

Survival and growth of stem cells are facilitated by single or a combination of growth factors viz. Epidermal Growth Factors (EGF), Fibroblast Growth Factor, basic (bFGF), Platelet-derived Growth Factor (PDGF) etc. For instance, bFGF is a member of heparin-binding growth factor family that induces stem cell proliferation at higher concentrations, while, inducing differentiation along with EGF at lower concentrations (18). Likewise, Sonic hedgehog (*Shh*), a major protein in the hedgehog signalling pathway modify the fate of NSCs based on a concentration gradient by activating homeodomain proteins, NKx2.2 and Pax6, which bind to DNA in a sequence-specific manner (19). Table 1 summarizes the role of major inducers of differentiation on cellular signalling pathways involved in the differentiation of neuroglia. Apparently, great level of variations exist in the action of growth factors as well as morphogens on a dazzling range of cell surface receptors in orchestrating signalling pathways towards this process. Besides, stem cell proliferation or differentiation is not just a function of a few signalling pathways or a set of genes, rather it’s an outcome of the dynamic interaction between a range of small molecules moderating the action of various transcriptional factors.

**Table 1:** Major signalling mechanisms in neuron and glia differentiation

Cell Types	Inducers / Growth Factors	Signalling Mechanisms	References
Neurons	Forskolin, Indomethacin	Increase in cAMP and activity of Protein Kinases A/B	28, 33, 36
	Neurotrophins, RA	Signalling through MAPK/ERK and PI3K/Akt activity	39, 40, 67
Astrocytes	Cytokines, Neurotrophins	Activation of MAPK/ERK and increase in JAK/STAT	50, 92, 95
	Cytokines, Notch	Induction of gp130 receptors for JAK/STAT activity	101, 102, 103
Oligodendrocytes	RA, Shh, Neurotrophins	Signalling through RA and p38 MAPK pathways	109, 110, 113
	Notch, Shh	Induction of transcription factors by notch and Shh	111, 112, 115
Schwann Cells	Neregulin-1, LPA	Increase in cAMP by activation of GPR44 and GPR126	118, 120
	Neurotrophins	Induction of specific transcription factors by PI3K/Akt	125, 126

*cAMP*, cyclic Adenosine monophosphate; *BMP*, Bone Morphogenetic Proteins; *GPR*, G Protein Coupled Receptors; *JAK-STAT*, Janus Kinases - Signal Transducer and Activator of Transcription (STATs); *LPA*, Lysophosphatidic acid; *MAPK/ERK*, Mitogen Activated Protein Kinases/Extracellular signal-Regulated Kinases; *PI3K/Akt*, Phosphoinositide-3-Kinase/Akt; *RA*, Retinoic Acid; *Shh*, Sonic hedgehog; *Wnt*, Wingless

### Cyclic AMP Signalling

**Fig. 1:**
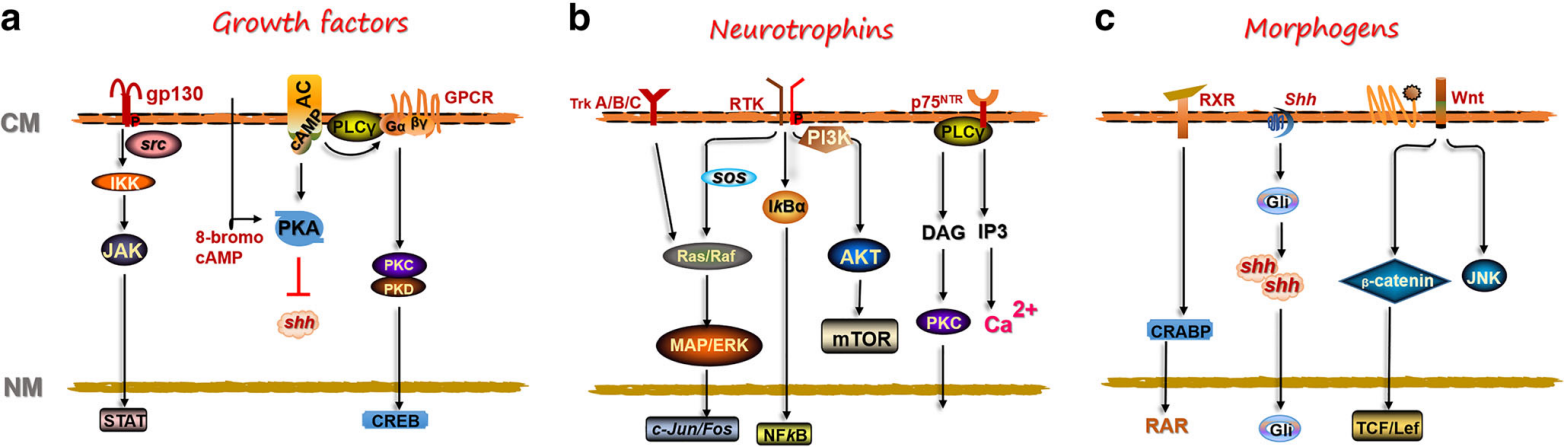
**Signal flow in stem cells for differentiation to neurons:** Constitutive signalling by growth factors and cytokines through transmembrane glycoprotein 130 (gp130) and G Protein Coupled Receptors (GPCRs) is essential for the maintenance of stemness, proliferation and differentiation (20, 21). Additionally, signalling by *Shh* should be abrogated to switch from stem cell proliferation to differentiation (**a**). Tyrosine Kinases (RTKs) signals through two important pathways viz. Phosphatidylinositol-3’-Kinase (PI3K), which is attributed to the maintenance and survival of stem cells during neural differentiation and Mitogen Activated Protein Kinases MAPK, which is responsible for the maturation of neuronal progenitors to neurons (41). Activation of PLCγ leads to generation of IP_3_ and DAG. The role of IP_3_ is the elevation of cellular Calcium levels while DAG activates signalling by PKC (40) (**b**). Further, stimulus from retinoic acid, *Sonic hedgehog* (*Shh*) and *Wingless* (Wnt) are critical for attaining neuronal morphology and neurite extension during differentiation (**c**). *Abbreviations: AC, Adenylate Cyclase; cAMP, cyclic Adenosine monophosphate; Ca*^*2+*^*, Calcium ions; CM, Cell membrane; DAG, Diacylglycerol; IP3, Inositol trisphosphate;JAK, Janus Kinase;* NM*, Nuclear membrane; NICD, Notch Intracellular Domain; P75*^*NTR*^*, Neurotrophin receptor P75; PLCγ,* Phospholipase Cγ; *PKC/D, Protein Kinase C/D; RXR, RAR, Retinoic Acid Receptor/complex*

Upon binding to bind to specific ligands, the membrane-bound G protein coupled receptors (GPCRs) undergoes conformational changes to activate adenylyl cyclase enzyme that catalyses the conversion of Adenosine Triphosphate (ATP) into cyclic adenosine monophosphate (cAMP) (20). Further, any changes in the microenvironment of MSCs by inducers cause mitochondria to generate Reactive Oxygen Species (ROS), releasing cAMP (Fig. 1a). This activates Protein Kinase A (PKA) affecting a wide variety of cellular processes including stem cell proliferation or differentiation (21, 22). Besides, several inducers signal through phosphatidylinositol 3-kinase (PI3K)/Akt (Akt is also called Protein Kinase B) pathway, which regulates MSC migration, proliferation or differentiation (23, 24). In developing brain, NSCs activate NADPH oxidase (NOX) upon stimulation by exogenous ROS, which regulate self-renewal and neurogenesis in a PI3K/Akt-dependant manner (24, 25). However, blockade of PI3K/Akt alone does not abrogate neuronal differentiation indicating the existence of other pathways for signal flow (26, 27).

Mitogen-activated Protein Kinases (MAPK) pathway consists of a chain of proteins, Ras-Raf-MEK-ERK in the cytoplasm that transmits signals from Receptor Tyrosine Kinases (RTKs) on cell surface to nucleus. Here, RTKs performs a Ras-dependent recruitment of Raf-1 for the phosphorylation and downstream signalling through MAPK pathway. Besides, MAPK regulates stem cell proliferation or differentiation through the mediators of cAMP signalling, Rap1/B-raf. Forskolin (a plant extract) phosphorylates PKA and a protein, B-raf in bone marrow MSCs by increasing the cellular levels of cAMP, leading to the activation of MAPK and neuronal differentiation (28). Therefore, the conditions that increase intracellular cAMP could differentiate MSCs into Neural Progenitors Cells (NPCs), often, without maturation into any specific lineages (29, 30). However, a rise in cAMP also activates phosphodiesterases (a cAMP inhibitor) and the use of phosphodiesterase inhibitors viz. dibutyryl-cAMP or 3-isobutyl-1-methylxanthine (IBMX) could be an alternate strategy for inducing neuronal differentiation (31, 32). Besides, 8-bromo-cAMP, a membrane-permeable cAMP derivative, resistant to phosphodiesterase, activates cAMP-dependent protein kinases in MSCs leading to proliferation or differentiation. Kompisch et al. reported an elevation of cAMP by combining IBMX with COX-2 inhibitor, indomethacin and a neuroprotectant, insulin (33). A prolonged effect of cAMP and activation of MAPK can be achieved by combining forskolin with growth factors (34, 35). Perhaps, forskolin with bFGF is a better combination for the phosphorylation of B-Raf and signal transduction by MAPK for neuronal differentiation (28, 36). Just as PKA, forskolin suppresses *Shh* illustrating the variable properties of inducers on signalling pathways (37, 38).

### Neurotrophin Signalling

Neurotrophins, such as brain-derived neurotrophic factor (BDNF), Nerve Growth Factor (NGF) and Neurotrophin (NT-3) along with the growth factors such as EGF, FGF, Platelet-derived Growth Factor (PDGF), Glia-derived Neurotrophic Factor (GDNF) and Vascular Endothelial Growth Factor (VEGF) mediate developmental neuronal differentiation. Neurotrophins bind to RTKs leading to endocytosis of receptor-neurotrophic complex initiating signal cascade for stem cell differentiation (Fig. 1b). They also signals through specific TrkA/B/C or the low-affinity p75^NTR^ receptors for the activation of cell surface Phosphoinositide phospholipase Cγ (PLCγ) and signal transduction through PI3K/Akt and MAPK/ERK pathways (39, 40). Activation of PKC by PLCγas well as small GTPases *Ras* and *Raf* releases calcium from the intracellular stores (40, 41). This stimulates signalling pathways, especially PI3K/Akt, which increases MSC survival and *Rac* activity (a member of the *Rho* family of GTPases) leading to changes in its shape and migration potential. Besides, polarization of *Rho*/myosin II components in response to growth factors alter cytoskeleton of differentiating cells, resembling fibroblast processes (42, 43). Additionally, binding of neurotrophins to a low-affinity receptor p75^NTR^, which is a member of the Tumor Necrosis Factor (TNF) receptor superfamily complements neurite extension (44, 45).

Differentiating ASCs upregulates neurotrophins receptors viz. NGFβR and NRP1 (Neuropilin 1, co-receptor of RTKs) upon stimulation by bFGF. Neurotrophin NGFβ promotes differentiation and NRP1 guides axon growth while the mitogenic bFGF enables cell survival during neurogenesis (46, 47). ASCs specifically express the necdin homolog (NDN) gene, which is associated with the NGFβ signalling for the glial migration during nervous system development (48). An increase in intracellular cAMP also increases the availability of Trk receptors in retinal ganglions, indicating a cross talk between two or more signal transduction pathways (49). However, the net result of this signalling cascade is also dependent on the type of stem cells used for differentiation (50). Lim et al., (2008) found that BDNF stimulates the neural differentiation of umbilical cord blood-derived MSCs by activation of MAPK/ERK and PI3K/Akt-dependent signalling pathways (51). Further studies proved that PI3K/Akt pathway facilitate survival of newly formed neurons, while MAPK/ERK enhance maturation upon stimulation by BDNF and bFGF, respectively (41).

### Wnt Signalling

Wingless (Wnt) proteins signals through seven-pass transmembrane receptor with β-catenin (canonical pathway) or without β-catenin (non-canonical pathway). These glycoproteins regulate neural patterning, axonal growth as well as synaptogenesis during embryonic development. They initiate transcription and cell proliferation in vitro by binding *Frizzled*/low density lipoprotein receptor-related protein (LRP) receptor complex leading to the release of β-catenin from glycogen synthase kinase-3β (Fig. 1c). Wnt signalling is proliferative for NPCs, although, it has a role in cell growth and differentiation of nervous system (52, 53). FGF (acidic) phosphorylates and deactivates glycogen synthase kinase-3β leading to the accumulation of β-catenin (54). β-catenin also promote proliferation of NSCs along with higher levels of bFGF, although, conditions like an early cell cycle exit may induce neuronal differentiation (55). Thus, β-catenins turns out to be neurogenic by activating basic helix-loop-helix (bHLH) family of transcription factors in stem cells (56).

Several Wnt proteins are identified and investigated for its cellular effects in biological models. Wnt1 upregulates T cell leukaemia 3 in MSCs for interacting with the TCF3/4 in the canonical pathway of Wnt signalling for neurogenesis (57). Role of Wnt3a is confirmed in differentiating MSCs by gene silencing (58). Further, neuronal differentiation of ASCs is mediated Wnt5a to signalling components *Frizzled* 3 or 5 through Wnt5α-c-Jun N-terminal kinase (JNK) pathway (59). Wnt signalling is also affected by changes in cellular redox status that diminishes the interaction of *Dishevelled* protein in Wnt pathway with other signalling components. In this case, binding of thioredoxin-like protein, nucleoredoxin to *Dishevelled* protein is inhibited by ROS, thereby activating Wnt/β-catenin pathway (60, 61). Conversely, conditions that inhibit release of calcium from intracellular stores lower ROS and the dissociation of *Dishevelled* protein from nucleoredoxin thereby attenuating Wnt/β-catenin signalling, compromising its pro-neural effects (62).

### Retinoic Acid Signalling

Retinoic acid (RA), a metabolite of vitamin A that signals by receptor translocation to nucleus regulating cell cycles in such a manner that switches stem cell proliferation to differentiation. RA enters into the cytoplasm of differentiating MSCs through its receptor RXR and binds to *cellular retinoic acid binding protein*. This complex enters the nucleus to bind retinoid specific receptors, which then bind to DNA sequences for transcriptional activity. RA has a generalized effect on human brain development and thus, aids in neural patterning and glial cell formation. In embryonic stem cells, RA along with neurotrophins, *Shh* and bFGF promote neuronal differentiation (63, 64). However, in MSCs a combination of RA and neurotrophins stimulates neurogenesis and synaptic induction with Wnt7a through canonical pathways. By contrast, speciation of these differentiating neurons is possible by activation of Wnt non-canonical/JNK pathways (65). Together, RA and bFGF downregulate Wnt as well as *notch* signalling in proliferating ASCs towards a neuronal phenotype (66, 67). Thus, the signal flow through bFGF, NGF, RA and *Shh* pathways seems to be advantageous for differentiation, indicating the similarities in signalling processes in vivo and in vitro (56, 67). Yet, how well these pathways are coordinated inside progenitor cells in favour of neurogenesis is intriguing.

Primary function of RA is to regulate the cell cycle to halt proliferation, thus redirecting cellular machinery to differentiation. In fact, the loss of RA signalling is associated with dedifferentiation and the development of cancer. Therefore, many of the differentiation protocols use RA alone or in combination with cell growth factors (67). In one instance, differentiation of ASCs to neurons is achieved by culturing them for a few days followed by seeding on a 24-well plate. Wells are added with the neural induction medium consisting of Dulbecco’s Modified Eagle’s Medium (DMEM) / F12 and N2 supplement (Merck) with 1% bFGF and 1% EGF (unpublished). ASCs treated with these mitogens as well as RA demonstrated neuronal morphology and markers of neural lineage cells (68, 69). Activation of MSCs with RA followed by forskolin facilitates neuronal differentiation of bone marrow MSCs, manifested as an increase in calcium and membrane potential (70). Perhaps, there is a signalling cross talk by cellular secondary messenger viz. cAMP or changes to cytoskeleton instigated by calcium ions (32). Investigations have to be conducted for the understanding the effect of RA differentiation with changes in ROS, often regarded as the tertiary messenger in stem cell signalling (71).

### Other Signalling Molecules

Other signalling molecules, Bone morphogenetic proteins (BMP) and Transforming Growth Factor-β (TGFβ) utilize intracellular SMAD proteins to regulate stem cell growth and differentiation. BMPs are classified into several subfamilies viz. BMP-2/4, BMP-5/6/7/8, growth and differentiation factor (GDF)-5/6/7, and BMP-9/10 groups. They also use the transforming growth factor β (TGFβ) receptor to regulate growth and differentiation through phosphorylation of the intracellular SMAD (a transcription factor) proteins (72). In embryos, BMP signalling is inactivated as the levels of chordin and noggin increases, which binds to the receptors of TGF-β super family. (73). Thus, BMP signalling inhibits the transformation of primitive ectoderm into neural ectoderm (74). And, abrogation of BMP signalling was found to improve the neural differentiation in ASCs (73). Yet, it has a positive influence in the regulation of dorsal neural cell types after initiation and formation of neural tissue (75).

*Notch* is a single spanning transmembrane protein that regulates the spatial patterning, timing and outcomes of many different cell fates during development (76). In embryos, *notch* signalling occurs by lateral inhibition, thereby a greater variation occur in the levels of transcriptional factors and their targets leading to suppression of neuronal differentiation (66). In fact, *notch* functions through the downstream bHLH proteins, Hes1 and Hes5 for maintaining NSCs in an undifferentiated state (77). However, following an increase in Sox21, bHLH proteins Mash1 and neurogenins induce an early cell cycle exit by limiting transcriptional factors Sox1/2/3 (78, 79). Therefore, *notch* has to be mitigated by activating transcriptional repressor, Prox1 by signalling through Wnt pathways for an effective neuronal differentiation (80, 81). Besides, this process increases *Shh* activity in embryos leading to asymmetric divisions of radial glia towards speciation of neuroglia (82).

### Differentiation of Glia

During embryogenesis, cell signalling pathways and transcription factors tightly regulate the switch from neurogenesis to gliogenesis. This process is dependent mainly upon the intrinsic timing and environmental cues (83). NSCs undergo asymmetric division to produce glial-restricted progenitors, which generate macroglia consisting of astrocytes and oligodendrocytes. During neurogenesis, these macroglia develop characteristic morphology in order to guide neuronal outgrowth, formation of blood-brain barrier and onset myelination of axons. On the contrary, microglia are the immune cells of the brain and their origin is elusive. Although, they are now speculated to be arising from the progenitors of embryonic yolk sac (84). In embryos, *Shh* and Wnt mediate dorsoventral patterning of the neural tube and compartmentalization of neurons and glia (85). Signalling through Wnt pathway is also responsible for neurogenesis and astrocyte differentiation, while, suppressing oligodendrocytosis (86, 87). Besides, BMP4 and Smad proteins aid in astrocyte differentiation by capsizing oligodendrocyte development (88, 89).

### Astrocytes

Astrocytes maintain the extracellular ion balance and biochemical support network of endothelial cells for maintaining the blood-brain barrier. Following a traumatic brain injury or an injury to the spinal cord several components of cellular and signalling pathways are activated leading to the proliferation and migration of NSCs and NPCs for self-repair by formation of ‘reactive astrocytes’ (90, 91). This is associated with glial scar formation that might inhibit axonal regrowth, although, over a period there will be neuronal and glial protection mediated by BDNF, ciliary neurotropic factor (CNTF), Interleukin (IL)-1β, IL-6, IL-11, Leukemia Inhibiting Factor (LIF) and NGF secreted by ‘reactive astrocytes’ (92). These cytokines and trophic factors along with bFGF coregulate Akt and ERK pathways in such a manner that Akt phosphorylation is inhibited, and the ERK pathway is activated by lysophospholipid ligands of GPCRs (50). Often, this is associated with the activation of JAK/STAT pathway and the accumulation of STAT3 for survival and formation of scar at the site of injury (93). Thus, there is a dynamic interaction between the components of inflammatory signalling with BMP and *notch* pathways in modulating the functional properties of ‘reactive astrocytes’ (94, 95).

**Fig. 2:**
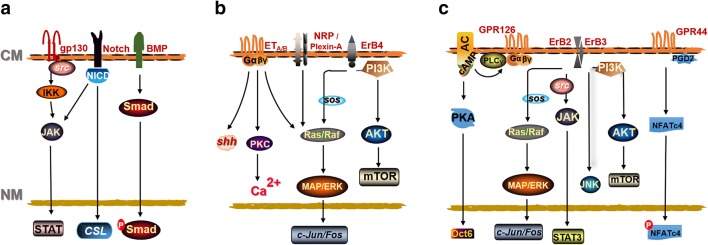
**Cell signalling for the differentiation of glia:** In NPCs, Wnt promotes neurogenic phase than the glial phase by upregulating neurogenins. Although, most of the processes are still obscure, growth factors along with Bone Morphogenic Proteins (BMP) and *notch* differentiates astrocytes in vitro (**a**). *Notch* mediates stem cell fate and precisely, inhibits oligodendrocyte specification (74, 154). Signalling through transmembrane Neurolipin (NRP) – Plexin A, Endothelin receptors (ET_A/B_) and neuregulin receptor (ErB4) are classical for oligodendrocyte development (**b**). GPCRs (GPR44 and GPR126) translocate transcription factor NFATc4 to nucleus and elevates cAMP for Protein Kinase A (PKA) signalling during Schwann cell differentiation and myelination (118). Moreover, ErB2/3 is a heterodimer transmembrane receptor for the binding of growth factors and activation of Mitogen Activated Protein Kinase (MAPK), Janus Kinase (JAK), c-*jun* N-terminal Kinase (JNK) and Phosphatidylinositol-3’-Kinase (PI3K) pathways (121, 126) (**c**). *Abbreviations: AC, Adenylate Cyclase; cAMP, cyclic Adenosine monophosphate; Ca*^*2+*^*, Calcium ions; CM, Cell membrane; NICD, Notch Intracellular Domain;* NM*, Nuclear membrane; PGD2, Prostaglandin D2; PLCγ,* Phospholipase Cγ; *PKC, PKD, Protein Kinase C/D*

In CNS, neurogenesis is followed by gliogenic switch, which engages several transcription factors for the regulation of genes driving astrocytogenesis (96). The neuronal cytokine, Cardiotrophin (CT-1) instructs NPCs to generate astrocytes under a negative feedback loop mechanism (97). In addition to forming a complex with CSL (a DNA binding protein) for astrogliogenesis, *notch* mediates translocation of N-CoR, which is a cofactor of the GFAP in the cytoplasm (98, 99). Further, signalling through glycoproteins 130 by LIF and CNTF followed by the activation of JAK/STAT is cardinal for the expression of GFAP markers in astrocytes (100, 101) (Fig. 2a). GPCRs can also promote astrocyte morphology and maturation by activation of cAMP pathways (102, 103). In rat pheochromocytoma cells (PC12), PKA signals through Rap1 and B-raf, which activates MAPK resulting in neuronal differentiation (104). Accordingly, an increased expression of B-raf is found in neurons but not in the astrocytes indicating the B-raf is the molecular switch for the differentiation of neurons and glia (105). cAMP activates MAPK in a Rap1/B-raf-dependent manner in neurons, but not in astrocytes. However, this is interfered by formation of a Rap1/Raf-1 complex in fibroblasts indicating the variations in signal flow between cell types (106).

### Oligodendrocytes

A sequence of events by neuralizing growth factors and metabolic hormones in the *milieu* of *Shh* signalling commence the formation of oligodendrocytes, which is responsible for insulating axons with myelin proteins and essential for signal conductivity in the CNS. Together, *Shh* and RA suppress the inhibitors specific for motor neurons leading to the activation of bHLH transcription factors Olig1 and 2 for the induction of oligodendrocyte progenitor cells (Fig. 2b). However, a gradual fading of Olig2 positive cells by RA and in the absence of *notch* leads to the maturation of oligodendrocytes into motor neurons (107). Thus, RA seems to be an essential component in methodologies for differentiating NSCs to oligodendroglia. Darbinyan et al. (2013) has shown in vitro differentiation of oligodendrocytes by plating NSCs onto poly-D-lysine coated dishes with a medium containing one part of self-conditioned *Neuronal Stem Cell Medium* (NSCM) and another part of *Oligodendrocyte Medium* (OM) (108). In fact, OM contains several growth factors viz. bFGF, EGF, PDGF-AA, L-glutamine and N2 supplement (Merck), primarily for inducing MAPK signalling towards oligodendrocytosis and myelin-specific gene expression (109, 110).

Differentiation and maturation of oligodendrocytes are controlled by distinct sets of transcription factors in forebrain and spinal cord. The transcription factors Olig1 and Olig2 of bHLH family are linked to the development of oligodendrocytes (111). The inception of promyelinating oligodendrocytes relies primarily on the homeodomain transcription factors, Nkx6.2 and Nkx6.2 as well as zinc finger proteins, Brn1 and 2. Another homeodomain protein Nkx 2.2, which is induced by *Shh* is also responsible for the oligodendrocyte maturation. Additionally, the decline of zinc finger protein, Krox24/Egr1 and transcriptional repression of E2F1 and *c*-Myc by a cyclin-dependent kinase (cdk) inhibitor, CDKN1B, facilitate an early cell cycle exit (100, 112, 113). The HMG domain proteins, Sox8/9/10, in combination with *notch* ligands Delta A and Delta B regulates speciation and terminal differentiation of oligodendrocytes (114). The expression of Sox4 and Sox11 also dictate the levels of POU domain proteins, Tst-1/Oct6/SCIP in oligodendrocyte precursors, which gradually decline upon maturation. Thus, induction of Sox17 or transduction of NSCs with Sox10 is the method adopted for generating oligodendrocytes in vitro (113, 115, 116).

### Schwann Cells

Differentiation of MSCs to Schwann cell lineage can be initiated by increasing cAMP signalling through GPCRs (specifically GPR126) in the presence of growth factors such as bFGF and PDGF acting on RTKs (117, 118). Both GPCRs - GPR126 and GPR44 elevate cAMP and activate the transcription factor Nfatc4 in favour of Neregulin 1-induced differentiation (119, 120) (Fig. 2c). β-heregulin, which is a neuregulin-1 anchors ErbB3/4 on RTKs and signals through PI3K/Akt pathway during the early stages of Schwann cell differentiation (121). Besides, LIF and Lysosphospatidic Acid (LPA) engages MAPK/ERK pathway for the survival of MSCs in differentiating cultures (122, 123). LIF along with bFGF coregulate Akt and ERK in such a manner that Akt phosphorylation is inhibited and the MAPK/ERK pathway is activated by signals originating from these lysophospholipid ligands through GPCR. Net result of this signalling cascade towards differentiation or proliferation is again dependent on the ligand activation and target cell types used (50).

During embryogenesis, a set of POU domain protein Tst-1/Oct6/SCIP and zinc finger protein Brn2 initiate the differentiation of precursor cells into promyelinating Schwann cells. A paired homeodomain protein, Pax3 is involved in the myelination of these precursor cells, while facilitating the dorsoventral patterning of the neural tube (112). Further, Sox10 is involved in the differentiation as well as activation of Pax3 and Tst-1/Oct6/SCIP for myelination (124). This is followed by the expression of zinc finger protein, Krox20/Egr2, which is attributed to the decline of POU protein during the terminal stages of Schwann cell differentiation (112). Thus, signalling through PI3K/Akt pathway is essential for the stimulation of these transcription factors in differentiation and myelination of Schwann cells (112, 125). During radial sorting, these immature Schwann cells gets polarized by Rho GTPases and laminin, which either spiral their membrane around neuronal axons to form myelin sheath or remain as unmyelinated Remak bundles (126).

## Neurospheres

Neural induction occurs by the generation of neural “rosettes”, which morphologically represent the neural tube. NPCs lodged in these neural “rosettes” are the source for the origin and development of neurons and glia. So, the differentiation of NPCs in cell culture can be performed by subjecting them to small molecules of growth that will mimic the developmental cues of brain during organogenesis (127). Upon seeding in *neurosphere differentiation medium*, MSCs begin to proliferate forming small clusters that detach from the culture surface and grow in suspension (Table 2). These clusters are differentiated by seeding in four-well plate (Nunc) in culture medium without EGF and, with tapering concentration of bFGF (i.e. EGF withdrawal) for 2–3 days (18, 128). Here, signalling by ligands of *tyrosine kinase* receptors play a role in the survival and differentiation of ASCs. Alternatively, neurospheres are differentiated in *neuronal differentiation medium* using dibutyrylcAMP for stimulating cAMP signalling pathway. Further, neuronal differentiation is initiated by plating these spheres on poly-L-ornithine or laminin-coated coverslips or chamber slides for attaching and dispersing without overlap (129). They can also be seeded on a 6-well poly-D-lysine coated tissue culture plate (45) or poly-D-lysine-coated BioCoat 8-well culture slide (130) with *neurobasal medium*.

**Table 2:** Composition of culture media for neuron and glia differentiation

Culture Medium	Composition	References
Neurobasal medium	Neurobasal medium is a basal medium to meet the special requirements of neuronal cells	(152)
Neurosphere growth medium	Neurobasal medium supplemented with 20 ng/ml EGF, 20 ng/ml bFGF, 2 mM L-glutamine and 2% StemPro neural supplement	(129)
Neurosphere differentiation medium	Neurobasal medium supplemented with 10% FBS and 2 mM L-glutamine	(129)
Neuronal differentiation medium	Neurobasal medium with 2% B27 supplement and 2 mM L-glutamine	(18)
Neural induction medium (Neurogenic medium)	α-MEM with L-glutamine containing 10% FBS, 0.5 mM IBMX, 1 ⌠M dexamethasone, 1% HEPES and 1% non-essential amino acids	(153)
Astrocyte differentiation medium	Neurobasal medium supplemented with 10% FBS, 100 ng/ml CNTF and 2 mM L-glutamine	(129)
Oligodendrocyte differentiation medium	Neurobasal medium with 2% B27 supplement, 30 ng/ml T3 and 2 mM L-glutamine	(129)
Schwann cell differentiation medium	DMEM/F12 with 10% FBS, forskolin, bFGF, PDGF and recombinant human heregulin-β1	(131)

*α-MEM*, Minimum Essential Medium Eagle, Alpha; *bFGF*, Fibroblast Growth Factor, basic; *CNTF,* Ciliary Neurotrophic Factor; *EGF*, Epidermal Growth Factor; *DMEM*, Dulbecco’s Minimal Essential Medium; *FBS*, Fetal Bovine Serum; *IBMX*, 3-isobutyl-1-methylxanthine; *PDGF*, Platelet-Derived Growth Factor

For obtaining astroglia, neurospheres are seeded into a 4-well plate (Nunc) in culture medium (without EGF or bFGF) and supplement with either 1% Fetal Bovine Serum (FBS) or BMP4. After two days of culture most of them will exit the cell cycle and acquire a characteristic stellate morphology and surface markers indicative of astrocytes (129). Alternatively, neurospheres can be differentiated in either *astrocyte differentiation medium* or *oligodendrocyte differentiation medium*. Culture dish is shaken overnight at 150 rpm in a 37 °C incubator to separate the adherent stellar cells (astrocytes) from the non-adherent glia cells (oligodendrocytes). For differentiating, Schwann cells from NSCs, neurospheres are triturated using a fire-polished Pasteur pipette and re-plated on laminin coated six-well chamber slides. These disoriented NSCs are maintained in DMEM/F12 supplemented with 10% FBS along with forskolin, PDGF, bFGF, recombinant human heregulin-β1 for a week. Here, differentiation into Schwann cells is achieved through RTKs including ErbB3 and 4 by heregulin-β1. Following two weeks of incubation, ASCs will differentiate into Schwann cells, expressing their characteristic phenotype at least or until 10 passages (131).

## Organoids

Organ-specific cell types from neural progenitors or neural rosettes can function as 3D culture models of nervous system in vitro. In embryonic brain, these structures evolve between cerebral cortex and choroid plexus expressing a homeobox transcription factor, LMX1A. Similar to signalling in developing forebrain, BMPs and Wnts are responsible for generating patterns on organoids in culture (132, 133). These cortical spheroids consist of neural progenitors that can develop into neurons or glia following treatment with bFGF and EGF. These cerebral organoids or cerebroids are independent bodies allowing the interaction between neurons and glia, often forming functional synapses (134). Astrocytes are differentiated from embryoids developed from human embryonic stem cells maintained in mTeSR1 medium (Stem cell Technologies) on Matrigel (BD Biosciences). Following mechanical disruption, these cerebroids are plated on polyornithine-laminin coated culture dishes and subsequently passaged in DMEM/F12 supplemented with 10% FBS (135).

Cerebral organoids can be generated in growth medium as suspended bodies or as static culture in Essential 8 (E8) medium using a hydrogel, termed Cell-Mate3D. They express not only gene and proteins of a developing brain but also an increase in intracellular calcium in response to elevated levels of glutamate and potassium (136). Organoids consists of multiple cell types that are spatially organized suitable for the modelling of developmental biology as well as replicating diseases and disorders of the host. Loss of function of PTEN-Akt signalling pathway identified a delay in neuronal differentiation but an increase in the proliferation of NPCs resulting in an increase size and folding of human cerebral organoids (137). Patient-derived cerebral organoids for Miller-Dieker syndrome identified defects in the expansion of radial glia due to disturbances in the N-cadherin/β-catenin signalling axis (138). More such initiatives have to be undertaken in future for understanding and establishing the implications of cellular signalling pathways in the development and organization of nervous system.

## Functionality

Generation of electrical activity during the differentiation of NPC is attributed to changes in the passive conductance by gap junctions and an increase in the intracellular chloride ions (139). There will be counter movements of Potassium (K^+^) and Chloride (Cl^−^) ions, which develop capacitance in accordance with the alternating current generated between these electrically coupled progenitor cells. An increase in the membrane potential arising due to this capacitance is stabilized by coupling of gap junctions, which lowers its input resistance (139, 140). In fact, astrocytes and oligodendrocytes communicate through these gap junctions, which is based on this passive movement of charges. Such an anatomical distribution and biochemical functionality of various ionic channels in neurons and glia could be instrumental in developing synaptic communications (140, 141). However, the evolution of ionic channels and neural excitability in NPCs need detailed investigation.

Just as in NPCs, glial cells tend to synchronize their calcium (Ca^2+^) oscillations for cell to cell communication, which is vital for its maintenance and proliferation. Besides, K^+^ channels can lower cell membrane potential required for the entry of calcium that facilitates cell cycle progression leading to mitosis and cellular proliferation (142). The immature glia facilitate the Na^+^ influx, which activates Na^+^-K^+^ pump as well as a functional Na^+^/Ca^2+^ exchanger to import calcium in exchange of sodium (143). Often, BMPs are capable of decreasing calcium spike activity necessary for differentiation and distribution of receptor and ionic channels. However, *Shh* signalling increases calcium resulting in the modulation of electrical activity, which is needed for the type of neurotransmitter release in spinal neurons (144, 145). Glial cells, especially astrocytes adopt this strategy for communication by which different signalling patterns tunes up specific neurotransmitter release evoking calcium currents (146). However, the important question here is the how much does cell signalling has an influence on the ionic channels during the process of differentiation.

## Challenges

Several experimental procedures using single or combination of growth factors have been adopted for the differentiation of MSCs to neuroglia. These ligands engage various cell surface receptors and signalling pathways in a variety of different ways, often crosstalk leading to an unexpected biological outcome. Chemical inducers give a faster rate of differentiation with neuron-like bodies, often without neuronal markers and functions (147). Thus, many of these neuronal differentiation protocols result in transient changes in the gene expression profile with morphological changes, without a clear distinction of neuronal functionality. Electrophysiological analysis is able to show the capabilities of newly formed nerve cells to carry electrical charges only in a few trails. However, these assays do not investigate the establishment of neuronal polarity and functional synapses (148). Thus, mostly the differentiation that we achieve in culture is only neuronal-like cells barely able to generate action potentials and form synapsis with adjacent or overlying cells. Besides, some of the protocols for the differentiation of neuroglia have ended in de-differentiation or reversion to stem cell features in the absence of constant stimulation (149, 150). Yet, these limitations should not discourage us from continuing to develop new methodologies for the differentiation of neuroglia that could eventually treat patients who are in need.

## Perspectives

The ability of isolated stems cells to cross barriers and differentiate into other dermal layers remains as a puzzle and challenge in regenerative medical science. Researchers are keen in elucidating the mechanistic role of various inducers on stem cell differentiation. However, there is a disconnect in our understanding on the signalling processes from surface receptors down to the nucleus for the assembly of transcriptional factors. Upon stimulation, differentiating cells can form a cluster of NSCs called neurospheres or an independent functional unit called organoid. These spheroids when grown in appropriate media and conditions will differentiate into a range of functional neuroglia. Thus, the role of paracrine factors and cellular microenvironment consisting of cell-cell communications has to be examined during differentiation (151). Unlike other cell types, differentiated neurons have to exhibit polarity, possess excitability to fire action potentials for impulse transmission. Further, they have to connect and communicate with various other cell types and tissues for releasing neurotransmitters at synapses. These criteria’s have to be fulfilled in vitro before using differentiated neurons and glia for any replacement therapies in vivo*.*
